# A Longer Duration of Intravenous Antibiotic Treatment for Patients with Early Periprosthetic Joint Infections Is Not Associated with a Lower Failure Rate

**DOI:** 10.3390/antibiotics14010079

**Published:** 2025-01-13

**Authors:** Janneke Meijer, Alex Soriano, Wierd Zijlstra, Bas ten Have, Saad Tarabichi, Paul Jutte, Javad Parvizi, Marjan Wouthuyzen-Bakker

**Affiliations:** 1 Department of Medical Microbiology and Infection Prevention, University Medical Center Groningen, University of Groningen, Hanzeplein 1, 9713 GZ Groningen, The Netherlands; j.meijer02@umcg.nl; 2 Department of Infectious Diseases, Hospital Clinic of Barcelona, University of Barcelona, 08036 Barcelona, Spain; asoriano@clinic.cat; 3 CIBERINF Ciber in Infectious Diseases, 28029 Madrid, Spain; 4 Department of Orthopaedic Surgery, Medical Center Leeuwarden, Henri Dunantweg 2, 8934 AD Leeuwarden, The Netherlands; wierd.zijlstra@mcl.nl; 5 Department of Orthopaedic Surgery, Martini Hospital, Van Swietenplein 1, 9728 NT Groningen, The Netherlands; havetenb@mzh.nl; 6 Department of Orthopaedic Surgery, Rothman Institute, Thomas Jefferson University Hospital, Philadelphia, PA 19107, USA; saad.tarabichi@rothmanortho.com; 7 Department of Orthopaedic Surgery, University Medical Center Groningen, University of Groningen, Hanzeplein 1, 9713 GZ Groningen, The Netherlands; 8 International Joint Center, Acibadem University Hospital, 34303 Istanbul, Turkey; javadparvizi@gmail.com

**Keywords:** periprosthetic joint infection, antibiotics, intravenous, oral, DAIR

## Abstract

Background: In recent years, many studies have demonstrated the efficacy of an early switch to oral antibiotics after surgical treatment in orthopedic-related infections. However, large analyses on periprosthetic joint infections (PJIs) are lacking. Material and Methods: We conducted a retrospective observational multicenter study in patients diagnosed with an early post-operative PJI, defined as one occurring <3 months after the index arthroplasty and treated with debridement, antibiotics, and implant retention (DAIR). Patients from Europe and the USA were included. We took advantage of the fact that an early oral antibiotic switch is routine practice in Europe as opposed to a long duration of intravenous (IV) antibiotic treatment in the USA. Failure was defined as the clinical need for (i) a second unintended DAIR procedure, (ii) implant removal, (iii) suppressive antibiotic treatment, or (iii) PJI-related death, all within one year after DAIR. Results: A total of 668 patients were included. A total of 277 received IV antibiotics for <14 days, 232 between 14 and 27 days, and 159 for >27 days. The overall 1-year failure rate within the 3 groups was 41.5%, 44.4%, and 42.1%, respectively (*p* = 0.80). This observation remained when excluding patients who failed during IV therapy. A longer duration of IV therapy seemed beneficial for those patients with a high pre-operative C-reactive protein level and lack of modular component exchange. Conclusions: In early post-operative PJIs, a longer duration of IV therapy is not associated with a lower failure rate but may be continued until a sufficient bacterial load reduction has been achieved.

## 1. Introduction

Periprosthetic joint infection (PJI) is one of the most serious adverse events after joint replacement and occurs in 1–9% of primary joint arthroplasties [1]. Provided that the implant is stable, early post-operative PJIs (occurring within three months after the index arthroplasty) can be treated with surgical debridement, antibiotics, and implant retention (DAIR) [2]. The recommended antibiotic treatment duration is 3 months in patients managed with DAIR [3]. However, the duration of intravenous antibiotics before switching to an oral alternative is still a controversial topic. Historically, IV therapy is believed to be superior to oral antibiotics because of better bioavailability and bone penetration [4]. However, in recent years, multiple studies have shown that an early switch to oral therapy, as opposed to continuing IV antibiotics, is safe and does not compromise the outcome [5,6,7,8,9,10]. In addition, oral antibiotics have fewer adverse events and reduce hospital stay in comparison to IV-only therapy [11,12]. In 2019, the OVIVA trial was published as a landmark paper supporting an early oral switch [6]. This multicenter randomized trial enrolled 1054 patients who were surgically treated for a bone and joint infection and were randomly assigned to an early oral switch (i.e., within 7 days after surgery) or a long duration of at least 4 weeks of IV therapy. The finding of the study was that oral antibiotic therapy was noninferior to IV antibiotic treatment. Although the findings of the OVIVA study were encouraging, extrapolation of these results to patients with a PJI who are treated with DAIR should be performed with caution. In the OVIVA trial, only 24% of included patients had osteosynthesis material and were treated with DAIR. The presence of biomaterials in PJI may influence the antibiotic effectivity and thereby the results of treatment. Thus, the current multicenter observational study, including centers from Europe and the United States of America (USA), was conducted to evaluate the efficacy of an early oral switch versus a long duration of IV antibiotics after DAIR. We took advantage of the fact that a long duration of IV antibiotics is common practice in the USA [13], while an (earlier) oral switch is a routine practice in Europe.

## 2. Results

### 2.1. Patient Characteristics

Data of 969 patients treated with a DAIR between 1999 and 2017 were evaluated. A total of 283 patients were excluded due to: (i) a follow-up of less than a year (n = 141), (ii) an infection in another location than the hip or knee (n = 5), (iii) a time interval of more than 90 days from index arthroplasty to DAIR (n = 113), (iv) the absence of positive cultures (n = 24), and (v) no data on duration of IV treatment (n = 18). Finally, a total of 668 patients were included, of whom 129 were from the USA and 539 from Europe.

From the total cohort, 388 (56.1%) patients were female, the mean age was 70 years (SD 11.9), and the mean body mass index was 31 kg/m^2^ (SD 6.4). A total of 425 patients had a PJI of the hip (61.4%), and 261 patients had a PJI of the knee (37.7%).

A total of 277 patients received IV antibiotics for <14 days (short IV group, 40.3%), 232 were given IV antibiotics between 14 and 27 days (intermediate IV group, 33.8%), and 159 received IV antibiotics for >27 days (long IV group, 23.2%). The median duration of IV therapy in the long IV group was 42 days (range 28–145 days). A total of 11.6% of patients from the USA cohort were in the short IV group, 7.0% in the intermediate, and 81.4% in the long IV group. Within Europe, this distribution was 48.6%, 41.4%, and 10.0%, respectively.

### 2.2. Patient, Implant, Surgical, and Microbiological Characteristics

Overall, the clinical characteristics between the three groups were comparable, but some differences were observed (Table 1). The patients who received a longer duration of IV antibiotics more often had a PJI of the hip, were more obese, and had a higher pre-operative serum CRP. Also, modular components during DAIR were less often exchanged. *Staphylococcus epidermidis* was less frequently isolated in the long IV group compared to the other two groups.

### 2.3. Failure Rates According to Duration of IV Antibiotics

**Figure 1:**
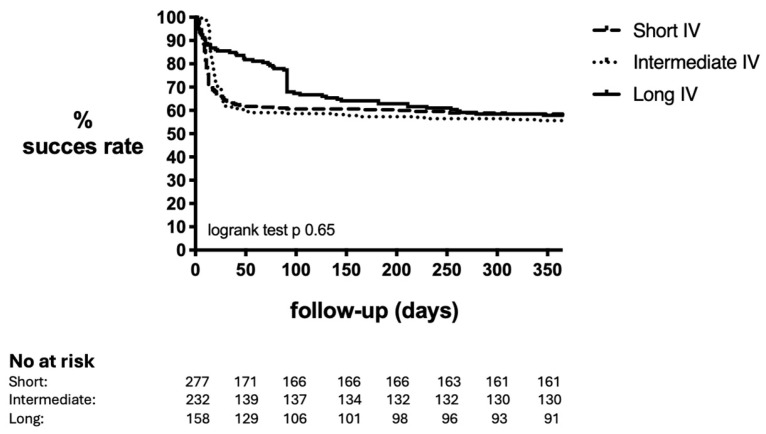
Success rate of DAIR within 1 year according to duration of IV antibiotics. IV: intravenous. Short: <14 days. Intermediate: 14–27 days. Long: >27 days.

Overall, 285 patients (42.7%) in our study had treatment failure 1 year after DAIR. From the failures, 239 patients (84%) failed during antibiotic treatment (i.e., within 90 days after DAIR) and 46 patients (16%) failed after finishing antibiotic treatment. The most common endpoint of failure was the need for a second unintended DAIR in 173 patients (60.7%), followed by implant removal in 79 patients (27.7%), long-term antibiotic suppressive therapy in 22 patients (7.7%), and PJI-related death in 11 patients (3.9%). A longer duration of IV treatment was not associated with a higher success rate. The overall 1-year failure rate within the short, intermediate, and long IV groups was 41.5%, 44.4%, and 42.1%, respectively (*p* = 0.80) (Table 2, Figure 1). Patients in the short IV group had a failure rate of 33.3% (39/117) when treated with IV for shorter than 7 days as opposed to 47.5% (76/160) when treated between 7 and 14 days (*p* = 0.018). We additionally analyzed failure defined as implant removal during a 1-year follow-up period. Implant removal amongst the 3 groups was 19.5%, 15.9%, and 15.7%, respectively (*p* = 0.47).

Table 3 shows the predictors of DAIR failure according to the univariate and multivariate analysis. Independent predictors for failure were fracture being the indication for arthroplasty (OR 2.4, 95% CI 1.47–3.88), a CRP >115 mg/L at initial presentation (OR 3.7, 95% CI 2.54–5.36), and the presence of *S. aureus* (OR 1.59, 95% CI 1.08–2.34).

### 2.4. Failure Rates Subgroups According to Duration of IV Antibiotics

We performed sub-analyses to evaluate whether a longer duration of IV antibiotics might be associated with a higher success rate in certain subgroups (Table 2). Obese patients did not benefit from a longer duration of IV antibiotics. Except for patients with renal insufficiency, no other comorbidities were associated with a higher success rate when treated with a longer duration of IV antibiotics. Interestingly, a higher failure rate was observed in knees when treated longer with IV antibiotics, while no difference was observed for hips. Additional analyses revealed that modular component exchange was performed more often in knees compared to hips (61.5% versus 38.5%, *p* < 0.001). Patients in whom modular components were not exchanged during DAIR had a higher success rate when treated with IV antibiotics longer. The same trend was observed for patients with a high pre-operative CRP, defined as a serum CRP level of more than 115 mg/L according to the KLIC-score as a risk factor for DAIR failure [14]. These patients also benefited from a longer duration of IV antibiotics of at least 14 days. This benefit of a longer duration of IV antibiotics in patients with a high pre-operative serum CRP and lack of modular component exchange remained after excluding patients who failed during IV therapy. Implant removal in patients with a high pre-operative CRP was 37.5% (30/80) in the short, 22.2% (16/63) in the intermediate, and 27.8% (14/66) in the long IV antibiotics group (*p* = 0.046).

We did not find any benefit of a longer duration of IV antibiotics based on the causative microorganism.

## 3. Materials and Methods

### 3.1. Patient Cohort

We conducted a retrospective multicenter observational study in which patients diagnosed with an early post-operative prosthetic joint infection (PJI) of the hip or knee and treated with debridement, antibiotics, and implant retention (DAIR) between 1999 and 2017 were included. Four centers from Europe and one center in the USA contributed patients to this study. We took advantage of the fact that an early oral antibiotic switch is a routine practice in Europe while a long duration of intravenous (IV) antibiotic treatment is custom in the USA [13].

PJI was defined according to the MSIS criteria [15]. Early post-operative PJI was defined as one that developed in less than 3 months after the index arthroplasty [2]. Patients without positive cultures, those with infections of joints other than hips or knees, and patients with a follow-up of less than one year (unless failure occurred before that timepoint) were excluded. Failure was defined as the clinical need for: (i) a second unintended DAIR, (ii) implant removal, (iii) suppressive antibiotic treatment, or (iv) infection-related death, all within one year after DAIR. All patients were treated with at least 3 months of antibiotics. For patients who failed due to the clinical need of suppressive antibiotic treatment, the endpoint of failure was set on 91 days (the day after finishing the “curative” antibiotic treatment approach).

### 3.2. Statistical Analysis

The duration of IV antibiotic treatment was divided into three groups (short: <14 days of IV antibiotic treatment; intermediate: 14–27 days of IV antibiotic treatment; and long: >27 days of IV antibiotic treatment). These cut-offs were chosen because in Europe, <14 days of IV therapy is common, and in the US, a minimum duration of 6 weeks is required. Patient characteristics, implant characteristics, surgical and microbiological findings were described according to the duration of IV treatment. A Kaplan–Meier curve was performed to analyze the failure rate according to the IV duration over time.

As IV therapy is often prolonged in cases with a poor clinical response after DAIR (e.g., persistent CRP and/or prolonged wound leakage), we performed a sub-analysis on failure after excluding patients who failed during IV therapy.

Categorical variables were expressed in percentages and absolute frequencies and were compared using the Chi-square test. Continuous variables were expressed as a mean and standard deviation and compared using the one-way ANOVA test. Variables associated with a higher failure rate, defined as a significance of *p* value < 0.1, were included in a multivariate analysis/logistic regression model using the enter method. Statistical analyses were performed using IBM SPSS Statistics (version 28).

## 4. Discussion

In this multicenter, multinational cohort study, we evaluated the clinical outcome of patients with an early post-operative PJI treated with DAIR. Overall, we demonstrated no difference in failure rate between a short (<14 days), intermediate (14–27 days), and long (>27 days) duration of IV antimicrobial treatment. This finding remained when reducing the IV duration to a maximum of 7 days, when excluding patients who failed during IV therapy, and when using implant removal as the primary endpoint of failure. However, we did observe a benefit from a longer duration of IV therapy (≥14 days) in patients with a high pre-operative serum CRP (>115 mg/L) and insufficient debridement (i.e., lack of modular component exchange during DAIR).

This is the first observational study in a large cohort of patients with PJI demonstrating that a short duration of IV antimicrobial treatment is safe. Our results are in concordance with previous publications demonstrating the safety of oral antibiotic therapy in a wide range of bone and joint infections. Wald-Dickler et al. performed a systematic review in 2022 that included prospective controlled trials evaluating an early oral stepdown in various invasive bacterial infections [5]. Eight of the included randomized controlled trials (RCTs) specifically focused on bone and joint infections, evaluating a total of 1321 patients. Four of these trials included patients with orthopedic hardware [6,7,8,9,10], of which the OVIVA trial was the largest analysis, including 125 patients with PJIs [6]. All included RCTs in the meta-analysis demonstrated that oral antibiotics are non-inferior to IV antibiotics, and even one study demonstrated a superior effect of oral ofloxacin over IV beta-lactams, suggesting the importance of an early switch to biofilm-active agents [10].

As certain subgroups of patients may benefit from a longer duration of IV therapy, we performed additional subgroup analyses. Patients with obesity (BMI >30) did not benefit from a longer duration of IV antibiotics. We also did not find a lower failure rate of a longer duration of IV therapy based on the causative microorganism(s). We did observe that patients with chronic renal insufficiency had a lower failure rate when treated with IV therapy for a longer period compared to those who were treated with a shorter duration, which might be due to a compromised vascular state favoring the bioavailability of IV therapy. However, the number of patients with chronic renal insufficiency in our study was low (n = 46), which complicates the interpretation and clinical relevance of this finding.

Remarkably, patients with a PJI of the knee had a lower failure rate when treated with a shorter duration of IV therapy. Additional analyses revealed that this was probably due to the fact that modular components were more often exchanged in knees compared to hips. When modular components were not exchanged, a longer duration of IV therapy was associated with a lower failure rate. The same benefit of a longer duration of IV therapy was also observed for patients who presented with a high pre-operative serum CRP level. This observation may be explained by a high microbial burden in both scenarios in which IV therapy might be preferred in the initial period. The majority of bacteria are most likely in a planktonic and metabolically active state in cases of insufficient surgical debridement (by not exchanging the modular components) and a high serum CRP [16,17]. At this stage, administering a bactericidal antibiotic, like a beta-lactam, may be most important to achieve a rapid reduction in the bacterial load. When the serum CRP is sufficiently declined, bacteria embedded in biofilm remain. At this stage of infection treatment, administering an antibiotic active against in biofilm embedded bacteria is indicated. In general, oral antibiotics active against biofilm consist of fluoroquinolones for Gram-negative PJIs and fluoroquinolones or clindamycin combined with rifampicin for staphylococcal PJIs [18,19]. All these antibiotics exhibit excellent oral bioavailability.

Our study should be perceived in light of some limitations. First, although we took advantage of the fact that a long duration of IV therapy is common practice in the USA while a shorter duration of IV therapy is common practice in Europe, a direct comparison between both cohorts remains challenging. This is reflected in the heterogeneity amongst the three cohorts. Several differences between the short and long IV cohorts were present, and although these differences were not related to failure in our study, they might have influenced the results. In addition, a percentage of patients within Europe still received a long duration of IV therapy (i.e., more than 14 days). Since IV therapy is often prolonged in patients who already show clinical signs of failure, this could have introduced selection bias. However, when excluding patients who failed during IV therapy, an early oral switch was still not inferior compared to a longer duration of IV therapy. A second limitation of our study is the follow-up of only one year. Failures might have occurred afterwards. However, the vast majority of patients who fail after DAIR fail within the first year [18,20], and if an infection occurs afterwards, it is not always clear if these should be regarded as DAIR failure or a new infection. Third, detailed information on the type of IV and oral antibiotics was not available, which hampered the possibility to analyze the most optimal type and dose of oral antibiotics. However, fluoroquinolones for Gram-negatives and fluoroquinolones or clindamycin plus rifampicin for staphylococci are routinely prescribed in the participating European hospitals [21,22]. In addition, the type of oral antibiotic therapy was not considered a major determinant of treatment success in the OVIVA trial [6]. Finally, patients were retrospectively included within a time period of 18 years. Changes in clinical practice, including changes in antimicrobial treatment, within centers might have occurred within this time period.

In conclusion, a long duration of IV therapy is not associated with a lower failure rate in patients with an early PJI treated with DAIR. A minimum amount of 7 days of IV therapy seems sufficient. Our results do suggest that IV therapy should be continued for at least 14 days in patients with a high pre-operative serum CRP or lack of modular component exchange until a sufficient bacterial load reduction has been achieved. Future studies should focus on the speed of serum CRP decline after DAIR in relation to outcome and additional methods to improve the outcome of DAIR, including the use of local antibiotics, lysins, and/or bacteriophages [23,24,25,26].

## Figures and Tables

**Table 1:** Patient, implant, clinical, and microbiological characteristics according to duration of IV antibiotic treatment.

Variables	<14 Days IV Antibiotic Treatment (n = 277)	14–27 Days IV Antibiotic Treatment (n = 232)	>27 Days IV Antibiotic Treatment (n = 158)	*p* Value
** Patient characteristics **				
Age > 80 years	15.9% (44/277)	28.9% (67/232)	10.8% (17/158)	**<0.001**
Gender, male	46.2% (128/277)	37.1% (86/232)	48.1% (76/158)	**0.048**
BMI > 30	51.4% (127/247)	46.9% (97/207)	62.2% (97/156)	**0.013**
Diabetes mellitus	15.5% (43/277)	22.8% (53/232)	23.8% (30/159)	0.110
Chronic renal failure	5.4% (15/277)	9.5% (22/232)	5.7% (9/159)	0.154
COPD	18.4% (51/277)	17.2% (40/232)	17.0% (27/159)	0.991
Rheumatoid arthritis	5.8% (16/277)	6.9% (16/232)	7.5% (12/159)	0.752
** Implant characteristics **				
Type of arthroplasty–primary	85.1% (232/276)	81.0% (187/231)	80.1% (125/156)	0.312
Exchange modular components	48.7% (133/273)	31.6% (72/228)	38.2% (52/136)	**<0.001**
Hip	55.2% (153/277)	69.8% (162/232)	61.6% (98/159)	**0.003**
Indication of arthroplasty–Fracture	19.9% (55/276)	18.6% (43/231)	8.3% (13/156)	**0.005**
** Clinical characteristics **				
<6 wks from arthroplasty to DAIR	92.4% (210/227)	93.5% (217/232)	88.7% (141/159)	0.207
CRP > 115 mg/L *	30.0% (80/267)	27.8% (63/227)	43.4% (66/162)	**0.003**
** Microbiological characteristics **				
Polymicrobial infections	37.9% (105/277)	44.8% (104/232)	31.4% (50/159)	0.260
Gram-positive microorganisms	87.4% (242/277)	88.4 (205/232)	95% (151/159)	0.187
*Staphylococcus aureus*	44.4% (123/277)	44.8% (104/232)	57.9% (92/159)	0.14
*Staphylococcus epidermidis*	41.2% (114/277)	32.8% (76/232)	26.4% (42/159)	**0.006**
*Enterococcus species*	11.2% (31/277)	15.9% (37/232)	17.6% (28/159)	0.129
*Streptococcus species*	7.6% (21/277)	14.2% (33/232)	15.7% (25/159)	**0.015**
Gram-negative micro-organisms	29.6% (82/277)	26.7% (62/232)	16.4% (26/159)	**0.008**
*Escherichia coli*	8.7% (24/277)	6.9% (16/232)	3.8% (6/159)	0.152
*Pseudomonas species*	6.5% 17/262)	7.2% (16/223)	5.6% (3/54)	0.899
*Enterobacter cloacae*	6.5% (18/277)	4.3% (10/232)	1.3% (2/159)	**0.039**
*Proteus species*	5.1% (14/277)	6.0% (14/232)	4.4% (7/159)	0.764
*Candida species*	1.1% (3/277)	0.4% (1/232)	0.6% (1/159)	0.683

* The cut-off value for CRP was chosen based on the KLIC-score as a predictor of failure [14].

**Table 2:** Failure rate according to duration of IV antimicrobial treatment for all patients and according to subgroups of patients.

Variables	<14 Days IV Antibiotic Treatment	14–27 Days IV Antibiotic Treatment	>27 Days IV Antibiotic Treatment	*p* Value
** All patients **	41.5% (115/277)	44.4% (103/232)	42.1% (67/159)	0.798
** Subgroups Patient characteristics **				
Age > 80 years	68.2% (30/44)	52.2% (35/67)	47.1% (8/17)	0.169
Gender, male	45.3% (58/128)	53.5% (46/86)	44.7% (34/76)	0.424
BMI > 30	37.8% (48/127)	43.3% (42/97)	42.3% (41/97)	0.67
Diabetes mellitus	46.5% (20/43)	54.7% (29/53)	40.0% (12/30)	0.416
Chronic renal failure	73.3% (11/15)	59.1% (13/22)	22.2% (2/9)	**0.048**
COPD	51.0% (26/51)	47.5% (19/40)	48.1% (13/27)	0.940
Rheumatoid arthritis	62.5% (10/16)	43.8% (7/16)	41.7% (5/12)	0.453
** Implant characteristics **				
Type of arthroplasty–primary	41.3% (97/235)	40.1% (75/187)	39.2% (49/125)	0.925
Type of arthroplasty–revision	43.9% (18/41)	61.4% (27/44)	54.8% (17/31)	0.268
Knee	33.9% (42/124)	44.3% (31/70)	57.4% (35/61)	**0.009**
Hip	47.7% (73/153)	44.4% (72/162)	32.7% (32/98)	0.055
Indication of arthroplasty–Fracture	69.1% (38/55)	51.2% (22/43)	69.2% (9/13)	0.164
Exchange modular components	29.3% (39/133)	50.0% (36/72)	55.8% (29/52)	**<0.001**
No exchange modular components	52.9% (74/140)	41.0% (64/156)	34.5% (29/84)	**0.018**
** Clinical characteristics **				
<6 wks from arthroplasty to DAIR	42.2% (108/256)	43.8% (95/217)	40.4% (57/141)	0.820
≥6 wks from arthroplasty to DAIR	33.3% (7/21)	53.3% (8/15)	55.6% (10/18)	0.311
CRP > 115 mg/L *	80.0% (64/80)	61.9% (39/63)	53.0% (35/66)	**0.002**
** Microbiological characteristics **				
Polymicrobial infections	43.8% (46/105)	46.2% (48/104)	34.0% (17/50)	0.350
Gram-positive microorganisms	41.7% (101/242)	45.4% (93/205)	41.7% (63/151)	0.695
*Staphylococcus aureus*	52.8% (65/123)	47.1% (49/104)	52.2% (48/92)	0.657
*Staphylococcus epidermidis*	28.9% (33/114)	40.8% (31/76)	31.0% (13/42)	0.223
*Enterococcus species*	48.4% (15/31)	56.8% (21/37)	28.6% (8/28)	0.074
*Streptococcus species*	42.9% (9/21)	42.4% (14/33)	36.0% (9/25)	0.857
Gram-negative micro-organisms	40.2% (33/82)	46.8% (29/62)	42.3% (11/26)	0.734
*Escherichia coli*	50.0% (12/24)	50.0% (8/16)	33.3% (2/6)	0.748
*Pseudomonas species*	35.3% (6/17)	25.0% (4/16)	33.3% (1/3)	0.809
*Enterobacter cloacae*	33.3% (6/18)	40.0% (4/10)	50.0% (1/2)	0.866
*Proteus species*	50.0% (7/14)	57.1% (8/14)	28.6% (2/7)	0.462
*Candida species*	100% (3/3)	100% (1/1)	0% (0/1)	0.082

* The cut-off value for CRP was chosen based on the KLIC-score as a predictor of failure [14].

**Table 3:** Predictors of DAIR failure.

Variables	Univariate Analysis						Multivariate Analysis			
	Success (N = 383)	Failure (N = 285)	Crude Odds Ratio	95% CI Lower	95% CI Upper	*p* Value	Adjusted Odds Ratio	95% CI Lower	95% CI Upper	*p* Value
** Patient characteristics **										
Age > 80 years	14.4% (55/383)	25.7% (73/284)	**2.06**	1.40	3.05	<0.01	1.43	0.90	2.28	0.14
Gender, male	39.7% (152/383)	48.6% (138/284)	**1.17**	1.02	1.35	0.002	0.78	0.55	1.11	0.17
BMI > 30	52.8% (190/360)	52.4% (131/250)	0.99	0.71	1.36	0.927				
Diabetes mellitus	17.0% (65/383)	21.4% (61/285)	1.33	0.90	1.97	0.148				
Chronic renal failure	5.2% (20/383)	9.1% (26/285)	**1.82**	0.806	3.126	0.049	1.59	0.81	3.13	0.18
COPD	15.7% (60/383)	20.4% (58/285)	1.38	0.92	2.05	0.116				
Rheumatoid arthritis	5.7% (22/383)	7.7%(22/285)	1.37	0.74	2.53	0.309				
** Implant characteristics **										
Type of arthroplasty–primary	85.8% (326/380)	78.1% (221/283)	**0.59**	0.39	0.88	0.010	0.54	0.34	0.84	0.01
Exchange of modular components	41.8% (153/366)	38.4% (104/271)	0.87	0.63	1.19	0.383				
Hip	61.6% (2632/383)	62.1% (177/285)	1.02	0.74	1.40	0.898				
Indication of arthroplasty–Fracture	11.1% (42/380)	24.4% (69/283)	**2.60**	1.71	3.95	<0.01	2.39	1.47	3.88	**<0.001**
** Clinical characteristics **										
<6 weeks from arthroplasty to DAIR	92.4% (354/383)	91.2% (260/285)	1.17	0.67	2.05	0.574				
CRP (>115 mg/L) *	19.1% (71/371)	50.2% (138/275)	**4.26**	2.99	6.04	<0.01	3.69	2.54	5.36	**<0.001**
**Antibiotic treatment**										
<14 days IV–compared to longer	42.3% (162/383)	40.4% (73/285)	0.92	0.68	1.26	0.255				
**Microbiological characteristics ^#^**										
Polymicrobial infection	38.6% (148/383)	38.9% (111/285)	1.01	0.74	1.39	0.936				
*Staphylococcus aureus*	41.0% (157/383)	56.8% (162/285)	**1.90**	1.39	2.59	<0.01	1.59	1.08	2.34	**0.02**
*Staphylococcus epidermidis*	40.5% (155/383)	27.0% (77/285)	**0.55**	0.39	0.76	<0.01	0.83	0.55	1.27	0.396

* The cut-off value for CRP was chosen based on the KLIC-score (12). Variables with a *p*-value < 0.1 in the univariate analysis (marked bold) were entered into the multivariate model. ^#^ Only those microorganisms associated with failure in the literature were included in the univariate analysis.

## Data Availability

Due to privacy regulations, the raw data of the manuscript will not be made public.

## References

[1] Tsikopoulos K., Meroni G. (2023). Periprosthetic Joint Infection Diagnosis: A Narrative Review. Antibiotics.

[2] Löwik C.A.M., Parvizi J., Jutte P.C., Zijlstra W.P., Knobben B.A.S., Xu C., Goswami K., Belden K.A., Sousa R., Carvalho A. (2019). Debridement, Antibiotics, and Implant Retention Is a Viable Treatment Option for Early Periprosthetic Joint Infection Presenting More Than 4 Weeks After Index Arthroplasty. Clin. Infect. Dis..

[3] Bernard L., Arvieux C., Brunschweiler B., Touchais S., Ansart S., Bru J.-P., Oziol E., Boeri C., Gras G., Druon J. (2021). Antibiotic Therapy for 6 or 12 Weeks for Prosthetic Joint Infection. N. Engl. J. Med..

[4] Haddad N., Ajaz J., Mansour L., Kasemodel R., Jarvis J., Jarad J., Gorski H., Carr M. (2023). A Review of the Clinical Utilization of Oral Antibacterial Therapy in the Treatment of Bone Infections in Adults. Antibiotics.

[5] Wald-Dickler N., Holtom P.D., Phillips M.C., Centor R.M., Lee R.A., Baden R., Spellberg B. (2021). Oral Is the New IV. Challenging Decades of Blood and Bone Infection Dogma: A Systematic Review. Am. J. Med..

[6] Li H.K., Rombach I., Zambellas R., Walker A.S., McNally M.A., Atkins B.L., Lipsky B.A., Hughes H.C., Bose D., Kümin M. (2019). Oral versus Intravenous Antibiotics for Bone and Joint Infection. N. Engl. J. Med..

[7] Mader J.T., Cantrell J.S., Calhoun J. (1990). Oral ciprofloxacin compared with standard parenteral antibiotic therapy for chronic osteo-myelitis in adults. J. Bone Jt. Surg. Am..

[8] Gentry L.O., Rodriguez G.G. (1990). Oral ciprofloxacin compared with parenteral antibiotics in the treatment of osteomyelitis. Antimicrob. Agents Chemother..

[9] Euba G., Murillo O., Fernández-Sabé N., Mascaró J., Cabo J., Pérez A., Tubau F., Verdaguer R., Gudiol F., Ariza J. (2009). Long-Term Follow-Up Trial of Oral Rifampin-Cotrimoxazole Combination versus Intravenous Cloxacillin in Treatment of Chronic Staphylococcal Osteomyelitis. Antimicrob. Agents Chemother..

[10] Gomis M., Barberán J., Sánchez B., Khorrami S., Borja J., García-Barbal J. (1999). Oral ofloxacin versus parenteral imipenem-cilastatin in the treatment of osteomyelitis. Rev. Esp. Quimioter..

[11] Phillips M.C., Wald-Dickler N., Davar K., Lee R., Baden R., Holtom P., Spellberg B. (2023). Choosing patients over placebos: Oral transitional therapy vs. IV-only therapy for bacteraemia and infective endocarditis. Clin. Microbiol. Infect..

[12] Darley E.S.R., Bannister G.C., Blom A.W., MacGowan A.P., Jacobson S.K., Alfouzan W. (2011). Role of early intravenous to oral antibiotic switch therapy in the management of prosthetic hip infection treated with one- or two-stage replacement. J. Antimicrob. Chemother..

[13] Osmon D.R., Berbari E.F., Berendt A.R., Lew D., Zimmerli W., Steckelberg J.M., Rao N., Hanssen A., Wilson W.R. (2013). Diagnosis and Management of Prosthetic Joint Infection: Clinical Practice Guidelines by the Infectious Diseases Society of America.

[14] Tornero E., Morata L., Martínez-Pastor J., Bori G., Climent C., García-Velez D., García-Ramiro S., Bosch J., Mensa J., Soriano A. (2015). KLIC-score for predicting early failure in prosthetic joint infections treated with debridement, implant retention and antibiotics. Clin. Microbiol. Infect..

[15] Parvizi J., Gehrke T., International Consensus Group on Periprosthetic Joint Infection (2014). Definition of periprosthetic joint infection. J. Arthroplast..

[16] Lisboa T., Seligman R., Diaz E., Rodriguez A., Teixeira P.J.Z., Rello J. (2008). C-reactive protein correlates with bacterial load and appropriate antibiotic therapy in suspected ventilator-associated pneumonia. Crit. Care Med..

[17] Deckey D.G., Christopher Z.K., Bingham J.S., Spangehl M.J. (2023). Principles of mechanical and chemical debridement with implant retention. Arthroplasty.

[18] Beldman M., Löwik C., Soriano A., Albiach L., Zijlstra W.P., Knobben B.A.S., Jutte P., Sousa R., Carvalho A., Goswami K. (2021). If, When, and How to Use Rifampin in Acute Staphylococcal Periprosthetic Joint Infections, a Multicentre Observational Study. Clin. Infect. Dis..

[19] Rodríguez-Pardo D., Pigrau C., Lora-Tamayo J., Soriano A., Toro d., Cobo J., Palomino J., Euba G., Riera M., Sánchez-Somolinos M. (2014). Gram-negative prosthetic joint infection: Outcome of a debridement, antibiotics and implant retention approach. A large multicentre study. Clin. Microbiol. Infect..

[20] Wouthuyzen-Bakker M., Sebillotte M., Huotari K., Escudero Sánchez R., Benavent E., Parvizi J., Fernandez-Sampedro M., Barbero J.M., Garcia-Cañete J., Trebse R. (2020). Lower Success Rate of Débridement and Implant Retention in Late Acute versus Early Acute Periprosthetic Joint Infection Caused by Staphylococcus spp. Results from a Matched Cohort Study. Clin. Orthop. Relat. Res..

[21] Zijlstra W.P., Ploegmakers J.J.W., Kampinga G.A., Toren-Wielema M.L., Ettema H.B., Knobben B.A.S., Jutte P.C., Wouthuyzen-Bakker M., on behalf of the Northern Infection Network for Joint Arthroplasty (NINJA) (2022). A protocol for periprosthetic joint infections from the Northern Infection Network for Joint Arthroplasty (NINJA) in the Netherlands. Arthroplasty.

[22] Tornero E., Morata L., Martínez-Pastor J.C., Angulo S., Combalia A., Bori G., García-Ramiro S., Bosch J., Mensa J., Soriano A. (2016). Im-portance of selection and duration of antibiotic regimen in prosthetic joint infections treated with debridement and implant retention. J. Antimicrob. Chemother..

[23] Calanna F., Chen F., Risitano S., Vorhies J.S., Franceschini M., Giori N., Indelli P.F. (2019). Debridement, Antibiotic Pearls, and Retention of the Implant (DAPRI): A modified technique for implant retention in total knee arthroplasty PJI treatment. J. Orthop. Surg..

[24] Dudareva M., Kümin M., Vach W., Kaier K., Ferguson J., McNally M., Scarborough M. (2019). Short or Long Antibiotic Regimes in Orthopaedics (SOLARIO): A randomised controlled open-label non-inferiority trial of duration of systemic antibiotics in adults with orthopaedic infection treated operatively with local antibiotic therapy. Trials.

[25] Ferry T., Onsea J., Roussel-Gaillard T., Batailler C., Moriarty T.F., Metsemakers W.-J. (2024). Bacteriophage therapy in musculoskeletal infections: From basic science to clinical application. EFORT Open Rev..

[26] Sendi P., Ferry T. (2022). Lysins—A new armamentarium for the treatment of bone and joint infections?. J. Bone Jt. Infect..

